# CHARACTERISTICS OF HOSPITALS AND PROVIDER MARKETS ASSOCIATED WITH INCREASES IN HOME HEALTH CARE USE

**DOI:** 10.1093/geroni/igac059.2679

**Published:** 2022-12-20

**Authors:** Elizabeth Watts, Taylor Bucy, John McHugh, Dori Cross

**Affiliations:** University of Minnesota School of Public Health, Minneapolis, Minnesota, United States; University of Minnesota School of Public Health, Minneapolis, Minnesota, United States; Columbia University Mailman School of Public Health, New York, New York, United States; University of Minnesota School of Public Health, Minneapolis, Minnesota, United States

## Abstract

Many older adults require post-acute care from a nursing home or home health agency following hospitalization. Recent trends show providers are increasingly relying on home health agencies rather than institutionalized settings, with home health volume surpassing skilled nursing facility (SNF) volume since 2017. Using MedPAR patient encounter data from 2016–2019 and provider data from CMS, we analyze changes in the profile of patients receiving home health over time, showing that individuals discharged to home health are increasing in complexity based on hospital length of stay, comorbidities, and use of critical care services. Mixed effects models additionally suggest that grouping patients at the hospital and market level helps to account for unexplained variation in whether a patient is likely to receive home health versus SNF services. Examining the characteristics of hospitals and provider markets with increasing rates of home health referrals over time, we found that hospitals with increasing rates of discharge to home health were more likely to be for-profit facilities in urban areas with higher operating margins. However, this increase was not consistently tied to a corresponding decrease in rates of discharge to SNF, suggesting that hospitals are experiencing a combination of both patient-shifting across post-acute settings as well as an overall increase in baseline complexity of hospitalized patients over this time period. These results have implications for understanding the how policies currently being considered to improve value in the post-acute sector filter through heterogeneous market structures and complex organizational environments to impact patient care decisions.

